# Iron Metabolism in the Colorectal Tumor Microenvironment: From Preneoplastic Lesions to Cancer Progression

**DOI:** 10.3390/ijms27125318

**Published:** 2026-06-12

**Authors:** Anamaria-Vlăduța Tomoiagă, Șoimița-Mihaela Suciu, Cezara-Andreea Gerdanovics, Alexandru Gerdanovics, Mircea-Vasile Milaciu, Mirela-Georgiana Perne, Teodora-Gabriela Alexescu, Lorena Ciumărnean, Angela Cozma, Vasile Negrean, Simona Valeria Clichici, Olga Hilda Orășan

**Affiliations:** 1 Department of Internal Medicine, 4th Medical Discipline, “Iuliu Hațieganu” University of Medicine and Pharmacy Cluj-Napoca, Republicii Street, No. 18, 400015 Cluj-Napoca, Romania; vladutat@yahoo.com (A.-V.T.); andreea.ceza.irimie@elearn.umfcluj.ro (C.-A.G.); vasile.milaciu@umfcluj.ro (M.-V.M.); albmirela@yahoo.ro (M.-G.P.); teodora.alexescu@umfcluj.ro (T.-G.A.); angelacozma@yahoo.com (A.C.); vasile.negrean@umfcluj.com (V.N.); hilda.orasan@umfcluj.ro (O.H.O.); 2 Department of Physiology, “Iuliu Hațieganu” University of Medicine and Pharmacy Cluj-Napoca, Clinicilor Street, No.1-3, 400006 Cluj-Napoca, Romania; sclichici@umfcluj.ro; 3 Neurology Department, Clinical Rehabilitation Hospital, Viilor Street, No. 46–50, 400066 Cluj-Napoca, Romania; alexandru.gerdanovics@elearn.umfcluj.ro; 4 Neuroscience Department, “Iuliu Hațieganu” University of Medicine and Pharmacy Cluj-Napoca, Victor Babeș Street, No. 8, 400012 Cluj-Napoca, Romania; 5 Department 2, Faculty of Nursing and Health Sciences, “Iuliu Hațieganu” University of Medicine and Pharmacy Cluj-Napoca, Republicii Street, No. 18, 400015 Cluj-Napoca, Romania; lorena_ciumarnean@yahoo.com

**Keywords:** colorectal cancer, iron metabolism, iron homeostasis, hepcidin–ferroportin axis, labile iron pool, oxidative stress, tumor microenvironment, preneoplastic lesions, metabolic reprogramming, ferroptosis

## Abstract

Colorectal cancer (CRC) is a major global health burden characterized by progressive genetic and metabolic alterations, with iron metabolism being increasingly recognized as a key contributor to tumorigenesis. This review provides an integrated synthesis of current evidence on iron metabolism across the continuum of colorectal cancer development, from preneoplastic lesions to advanced disease. We analyzed data from epidemiological, experimental, and mechanistic studies addressing systemic and cellular iron homeostasis, including the hepcidin–ferroportin axis, as well as iron handling within tumor cells and the tumor microenvironment. Available data indicate that colorectal epithelial cells progressively develop an iron-retentive phenotype, characterized by increased iron uptake and reduced export, leading to expansion of the intracellular labile iron pool. This imbalance contributes to oxidative stress, DNA damage, metabolic adaptation, and activation of oncogenic signaling pathways while also influencing immune responses. However, epidemiological findings on dietary iron and CRC risk remain inconsistent, highlighting the context-dependent nature of iron-related effects. In conclusion, iron metabolism represents a dynamic regulator of CRC progression and a mechanistic framework for understanding stage-specific tumor evolution, although further studies are needed to clarify how iron-dependent pathways differ across colorectal tumor subtypes and microenvironmental contexts.

## 1. Introduction

Colorectal cancer (CRC) is one of the most common and lethal malignancies worldwide and represents a major global public health challenge, with nearly two million new cases diagnosed annually [1,2].

CRC develops through the progressive accumulation of genetic and epigenetic alterations that drive the transformation of normal colonic epithelium into invasive carcinoma through distinct molecular pathways, including chromosomal instability (CIN), microsatellite instability (MSI), and serrated neoplasia [3,4].

Iron is an essential micronutrient that supports multiple cellular processes critical for tumor development, including DNA synthesis and repair and redox homeostasis. Recent studies have expanded the understanding of iron metabolism in cancer by identifying novel regulatory mechanisms, including the role of nuclear receptor coactivator 4 (NCOA4) in ferritin turnover and iron homeostasis [5,6].

Beyond genetic instability and histopathological progression, metabolic reprogramming has emerged as a fundamental driver of CRC development. Tumor cells undergo a characteristic shift from oxidative phosphorylation to aerobic glycolysis (the Warburg effect), enabling rapid ATP generation and the production of biosynthetic intermediates that support proliferation under hypoxic and nutrient-deprived conditions [7,8,9]. This metabolic phenotype is regulated by oncogenic signaling pathways frequently altered in CRC, including MYC, RAS, and epidermal growth factor receptor (EGFR) signaling, which directly modulate glycolysis, glutaminolysis, and lipid metabolism [10,11].

Emerging evidence indicates that iron actively contributes to this metabolic phenotype by promoting glycolytic reprogramming. Excess intracellular iron enhances GLUT1 expression, inhibits pyruvate dehydrogenase (PDH)-dependent mitochondrial oxidation through PDHK3-associated mechanisms, thereby favoring aerobic glycolysis even under normoxic conditions. In addition, iron-mediated stabilization of HIF-1α further reinforces glycolytic gene transcription and metabolic adaptation [12,13]. Importantly, these pathways exhibit bidirectional interactions with iron metabolism. MYC upregulates transferrin receptor 1 (TfR1) and IRP2 while suppressing ferritin expression to increase intracellular iron availability, whereas EGFR-associated HIF-2α signaling promotes DMT1-mediated iron uptake, creating a feed-forward loop that sustains proliferation and metabolic adaptation [13,14].

Metabolic reprogramming extends beyond tumor cells and profoundly remodels the tumor microenvironment (TME), where lactate accumulation and local acidosis suppress antitumor immune responses and promote immune evasion by favoring regulatory T cells and M2-like tumor-associated macrophages [7,9,15]. In parallel, cancer-associated fibroblasts and tumor-associated macrophages adapt their metabolic programs to further sustain tumor growth and immune suppression [15]. Iron further shapes these interactions, as iron-loaded macrophages produce IL-6 and promote hepcidin-mediated iron retention, while excessive iron sequestration contributes to immune dysfunction and reinforces the immunosuppressive TME [16].

Epidemiological studies have suggested associations between elevated iron exposure, dietary iron intake, and CRC risk; however, available findings remain heterogeneous and sometimes contradictory, indicating that the biological effects of iron are highly context dependent [17]. This inconsistency likely reflects, at least in part, the difficulty of distinguishing between biologically distinct iron compartments: luminal iron directly contacts the colonic epithelium and promotes carcinogenesis through oxidative damage and microbiota-mediated mechanisms, whereas systemic iron influences tumor biology through transferrin-receptor-mediated uptake and intracellular redox perturbation [13,16]. Furthermore, systemic iron biomarkers yield paradoxical and inconsistent results, partly attributable to their behavior as acute-phase reactants and to reverse causation arising from CRC-associated chronic blood loss, with Mendelian randomization studies providing only suggestive, non-definitive evidence for a causal role of circulating iron in CRC [18,19,20].

Although several recent reviews have addressed individual aspects of iron biology in CRC, including dietary iron exposure, inflammation, oxidative stress, and ferroptosis, the dynamic evolution of iron metabolism throughout the continuum from preneoplastic lesions to advanced disease remains incompletely integrated. Furthermore, the role of iron as a mediator of communication between tumor cells and the surrounding microenvironment has received comparatively less attention. In this context, the present review aims to provide an integrated overview of iron metabolism in colorectal tumorigenesis, spanning the continuum from preneoplastic lesions to advanced CRC. We summarize current evidence on systemic and local iron homeostasis, examine the molecular mechanisms governing iron uptake, storage, export, and utilization in colorectal tumor cells, and discuss how iron-dependent processes interact with oxidative stress, metabolism, immune regulation, and the tumor microenvironment during CRC progression. Particular emphasis is placed on stage-specific alterations in iron handling, tumor-associated iron remodeling, and microenvironmental iron redistribution across different stages of colorectal carcinogenesis.

## 2. Literature Search Strategy

This narrative review was based on a comprehensive literature search conducted in the PubMed, Scopus, and Web of Science databases. Articles published up to January 2025 were identified using combinations of keywords including “colorectal cancer”, “iron metabolism”, “iron homeostasis”, “tumor microenvironment”, “ferroptosis”, “hepcidin”, “ferroportin”, “transferrin receptor”, “oxidative stress”, “gut microbiota”, and “preneoplastic lesions”. Original research articles, mechanistic investigations, experimental studies, epidemiological studies, and relevant review articles were considered. Priority was given to studies providing direct evidence on iron metabolism during colorectal tumorigenesis, iron-dependent interactions within the tumor microenvironment, and mechanisms linking iron dysregulation to cancer progression. Where direct human tissue data were available, these were prioritized over findings derived exclusively from experimental models; in areas where CRC-specific human evidence remained limited, data from animal models and cell-based studies were included and clearly identified as such. Additional references were identified through manual screening of bibliographies from selected publications.

## 3. Overview of Iron Metabolism in Human Physiology

### 3.1. Systemic Iron Homeostasis

Iron is a redox-active metal that can donate and accept electrons and therefore exists in two main oxidation states: ferric (Fe^3+^) and ferrous (Fe^2+^) iron. In humans, the daily iron requirement is estimated at approximately 25–30 mg, which is largely met through dietary intake. Iron circulates in the bloodstream predominantly bound to transferrin (Tf), a glycoprotein responsible for delivering iron to peripheral tissues. Under physiological conditions, transferrin-bound iron represents the major circulating pool; however, when transferrin-binding capacity is exceeded, non-transferrin-bound iron (NTBI) appears in the plasma. A particularly reactive fraction of NTBI is labile plasma iron (LPI), which can be taken up by non-hematopoietic cells and promote parenchymal iron accumulation and oxidative tissue damage [21,22].

Dietary iron is present in two principal forms: heme iron and non-heme iron. Heme iron is primarily derived from meat and poultry, whereas non-heme iron is mainly obtained from plant-based sources such as cereals and vegetables. Intestinal iron absorption occurs predominantly in the duodenum and primarily involves ferrous iron. Ferric iron is poorly absorbed and must first be reduced to the ferrous form by duodenal cytochrome B (DCYTB) reductase at the apical membrane of enterocytes [22,23]. Several uptake mechanisms contribute to iron absorption, including erythrophagocytosis and the transferrin–transferrin receptor system, with divalent metal transporter 1 (DMT1) representing the principal transporter responsible for ferrous iron entry into enterocytes [22].

Once internalized, intracellular iron is directed toward utilization, storage, or export depending on systemic requirements. Iron storage predominantly occurs in hepatocytes, where excess iron is safely sequestered within ferritin (FT). Cellular iron export is mediated mainly by ferroportin (FPN), the only known mammalian iron exporter, which facilitates iron release following its oxidation to the ferric form and subsequent entry into the circulation [24].

FPN is expressed on the basolateral membrane of duodenal enterocytes, macrophages, and hepatocytes and plays a central role in maintaining systemic iron homeostasis. Structurally, FPN consists of 12 transmembrane helices organized into two lobes [25]. Following export, ferrous iron is oxidized to ferric iron by oxygen-dependent ferroxidases and subsequently bound to transferrin for systemic transport [22,23]. In the absence or dysfunction of ferroxidases, iron export is impaired, leading to intracellular iron retention within ferritin.

### 3.2. Cellular and Intracellular Iron Handling

At the cellular level, iron uptake from the circulation is mediated by the transferrin–ferric iron complex through interaction with TfR1 expressed on the cell surface. Only iron-saturated transferrin, referred to as diferric transferrin, is recognized by TfR1 and internalized via endocytosis into endosomal compartments. Within endosomes, ferric iron is reduced to the ferrous form and transported into the cytosol via DMT1, where it enters the labile iron pool for metabolic utilization or storage, while transferrin is recycled back to the circulation [26].

Systemic iron export and circulation are tightly regulated to prevent both iron deficiency and iron overload, a process primarily governed by the hepcidin–ferroportin axis. Hepcidin, a peptide hormone predominantly produced by hepatocytes, functions as the central regulator of systemic iron homeostasis by controlling iron efflux from cells. Its biological activity is mediated through direct binding to FPN, the only known cellular iron exporter, which is expressed on the surface of duodenal enterocytes, macrophages, and hepatocytes. Upon hepcidin binding, FPN undergoes internalization followed by lysosomal degradation, resulting in inhibition of iron export into the circulation and a subsequent reduction in serum iron levels [27,28,29].

Hepcidin expression is tightly regulated by multiple physiological and pathological signals to ensure that iron absorption and mobilization from body stores are aligned with systemic demands. Hepcidin synthesis is upregulated in response to increased plasma and hepatic iron levels, as well as during inflammation, primarily through activation of the IL-6/STAT3 signaling pathway [27,28,30]. Conversely, hepcidin expression is suppressed under conditions of iron deficiency, hypoxia, or heightened erythropoietic activity, including erythroferrone-mediated signaling originating from the bone marrow [31,32]. This finely tuned feedback system maintains systemic iron balance by coordinating dietary iron absorption with iron recycling and storage.

## 4. Iron and Colorectal Carcinogenesis

Despite substantial supporting evidence, epidemiological studies examining the association between dietary iron intake and CRC risk have yielded inconsistent results, with several methodological and biological factors accounting for these discrepancies [33,34,35,36,37,38,39]. First, the failure to distinguish between iron forms represents the single most important source of inconsistency: meta-analyses consistently demonstrate that heme iron is positively associated with CRC risk (RR 1.08–1.12 per 1 mg/day increase), while total dietary iron and non-heme iron show null or inverse associations, as non-heme iron sources carry protective co-nutrients including fiber, folate, and calcium that independently reduce CRC risk [40,41,42]. Second, sex-specific differences in iron metabolism, attributable to menstrual iron losses and hormonal hepcidin regulation, produce divergent associations in men and women that are obscured in unstratified analyses; the EPIC cohort demonstrated that the heme iron–CRC association was present only in men, while the Iowa Women’s Health Study found a positive association specifically in postmenopausal women who consumed alcohol [43]. Third, serum ferritin behaves paradoxically as both an acute-phase reactant elevated by tumor-associated inflammation and a marker subject to reverse causation from occult tumor bleeding, which systematically lowers iron stores before clinical diagnosis, creating bidirectional associations that linear models cannot capture [20,44]. Fourth, transferrin saturation reflects circulating iron availability rather than stored iron and shows more consistent positive associations with CRC risk, with a Finnish cohort of 41,276 participants demonstrating a threefold increased CRC risk at TSAT > 60%, suggesting TSAT may be a more relevant biomarker than ferritin for CRC risk assessment [44,45]. Fifth, anatomical subsite heterogeneity is frequently overlooked: luminal iron predominantly contacts the proximal colon while systemic iron influences the distal colon, and pooling of subsites attenuates subsite-specific effects [46]. Sixth, gene–diet interactions involving HFE polymorphisms, where C282Y homozygosity confers a twofold increased CRC risk, and SNPs in 21 iron homeostasis genes create substantial interindividual differences in iron handling that most observational studies cannot detect [47,48]. Seventh, food frequency questionnaires estimate heme iron using fixed proportions of total meat iron rather than measuring actual heme content, introducing non-differential misclassification that attenuates risk estimates toward the null [36,40]. Finally, iron plays a paradoxical dual role in CRC biology, with both iron deficiency, impairing immune surveillance, and iron overload, promoting oxidative DNA damage, potentially increasing risk through a U-shaped dose–response relationship that linear regression models cannot capture. Resolving these inconsistencies will require studies that simultaneously measure dietary heme iron with detailed cooking and processing data, multiple serum biomarkers, iron homeostasis gene genotypes, and CRC subsite, stratified by sex and with sufficient follow-up to exclude reverse causation [16].

Beyond direct mucosal toxicity, unabsorbed dietary iron substantially remodels the colonic microbiome, representing a critical pathological mediator of iron-driven colorectal carcinogenesis. Excess luminal iron selectively promotes the expansion of Gram-negative Proteobacteria while depleting butyrate-producing commensals such as *Roseburia* and *Faecalibacterium prausnitzii*, resulting in persistent intestinal dysbiosis, chronic mucosal inflammation, and COX-2 upregulation [49]. Pathogenic bacteria harboring sophisticated iron acquisition systems, including siderophore-mediated chelation mechanisms, possess a competitive advantage over commensals in iron-replete luminal environments, thereby further amplifying dysbiotic shifts [50]. Mechanistically, excess luminal iron has been demonstrated to render the gut microbiota pathogenic, compromising epithelial barrier integrity and facilitating bacterial translocation into the mucosa; in response, epithelial cells upregulate secretory leukocyte protease inhibitor (SLPI), which paradoxically activates MAPK signaling and promotes colorectal tumorigenesis [51]. The ensuing depletion of butyrate-producing taxa further impairs barrier homeostasis, attenuates anti-inflammatory signaling, and relieves inhibitory constraints on oncogenic pathways, including Wnt and TGF-β. Collectively, these findings suggest that the biological consequences of dietary iron exposure may depend not only on iron dose and form, but also on the composition and functional capacity of the intestinal microbiota, thereby providing a potential explanation for the heterogeneous epidemiological associations between dietary iron intake and CRC risk [52].

Both iron overload and iron deficiency appear to exert adverse effects on different aspects of CRC pathogenesis. Data suggest that excessive iron intake and elevated body iron stores may increase CRC risk, although a direct causal relationship has not yet been definitively established [53]. Iron overload may arise from two distinct sources: exogenous iron derived from excessive dietary intake, which can directly affect colonic tissue via luminal exposure, and endogenous iron overload resulting from elevated systemic iron levels, which can damage target organs and cells. Under physiological conditions, only approximately 10% or less of ingested iron is absorbed, leaving a substantial proportion of dietary iron unabsorbed in the intestine and available for interaction with the colonic mucosa. Notably, studies investigating the relationship between iron overload and CRC incidence and progression have produced controversial results [54].

Anemia, particularly iron deficiency anemia (IDA), is a common clinical feature in patients with CRC, affecting approximately 40–60% of individuals at diagnosis. IDA frequently results from chronic occult blood loss due to tumor bleeding, impaired iron absorption, or reduced dietary intake [38,39,55]. Iron deficiency negatively impacts quality of life, treatment tolerance, and survival outcomes in CRC patients. In addition, chemotherapy-induced anemia further contributes to iron imbalance in this population. Although oral iron supplementation has historically been used as a convenient and cost-effective treatment for IDA, iron repletion strategies in CRC patients require careful consideration. Both insufficient iron availability and iron overload may have clinically significant consequences for tumor biology and patient outcomes, underscoring the need for a balanced and individualized approach to iron management in CRC [38].

## 5. Iron Metabolism in Preneoplastic Colorectal Lesions

Colorectal adenomas are key preneoplastic lesions in the adenoma–carcinoma sequence, and increasing evidence indicates that iron metabolism is already perturbed at this early stage of colorectal tumorigenesis. Rather than reflecting a uniform pattern, iron-related alterations in adenomas involve a dynamic interplay between systemic iron status, local epithelial iron handling, oxidative stress, and lesion-specific molecular pathways [56].

At the systemic level, circulating iron parameters demonstrate heterogeneous and sometimes opposing trends across adenoma populations. Based on epidemiological analyses, higher body iron stores, most commonly reflected by elevated serum ferritin, are associated with an increased risk of colorectal adenomas, with the strongest signals observed for right-sided lesions and in a dose-dependent fashion. In one case–control study conducted in human populations, adenoma risk rose significantly across ferritin strata, with adjusted odds ratios of 3.8 (95% CI 1.5–9.5) and 5.1 (95% CI 2.0–12.7) in the third and fourth quintiles, respectively, compared with the lowest quintile. These findings highlight the potential contribution of systemic iron excess to early colorectal neoplasia [45].

Conversely, patients with large or bleeding adenomas may exhibit features of iron deficiency, most plausibly due to chronic occult blood loss. This apparent paradox observed in clinical and epidemiological studies highlights the dual role of iron in colorectal tumorigenesis: systemic depletion may reflect lesion-related bleeding, whereas increased mucosal iron availability may facilitate neoplastic initiation and progression [45].

**Figure 1:**
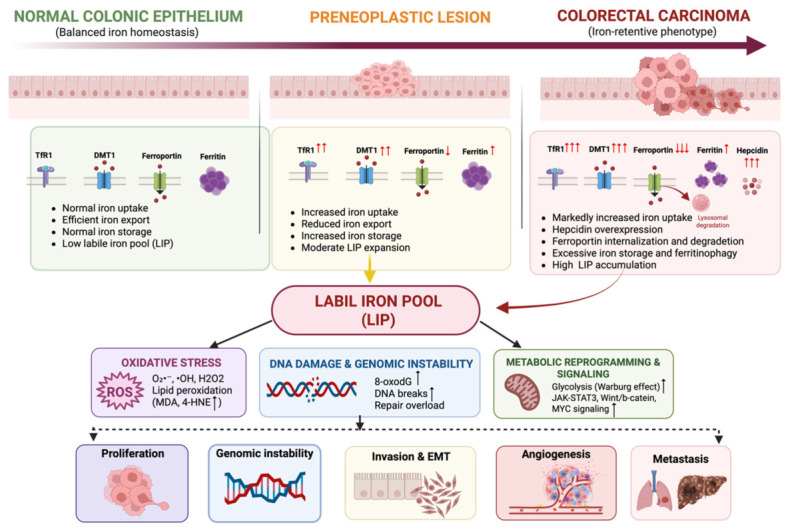
Progressive acquisition of an iron-retentive phenotype during colorectal tumorigenesis. Iron metabolism is progressively remodeled across the normal mucosa–preneoplastic lesion–carcinoma continuum: balanced import/export in normal epithelium gives way to increased TfR1- and DMT1-mediated uptake with impaired FPN-dependent export in adenomas, and to a pronounced iron-retentive phenotype in carcinoma characterized by marked TfR1/DMT1 upregulation, hepcidin-mediated FPN degradation, and ferritin accumulation. The resulting labile iron pool expansion drives ROS generation, oxidative DNA damage, oncogenic signaling (JAK/STAT3, MYC), metabolic reprogramming, EMT, and metastatic dissemination. Arrows indicate direction and magnitude of changes. This schematic represents a conceptual model that may not apply uniformly across all CRC molecular subtypes or clinical contexts. Abbreviations: TfR1, transferrin receptor 1; DMT1, divalent metal transporter 1; FPN, ferroportin, cellular iron exporter; LIP, labile iron pool; ROS, reactive oxygen species; O_2_•^−^, superoxide anion; H_2_O_2_, hydrogen peroxide; •OH, hydroxyl radical; MDA, malondialdehyde; 4-HNE, 4-hydroxynonenal; 8-oxodG, 8-oxo-7,8-dihydro-2′-deoxyguanosine; JAK, Janus kinase; STAT3, signal transducer and activator of transcription 3; MYC, MYC proto-oncogene; EMT, epithelial–mesenchymal transition. Created in BioRender. Vladuta, T. (2026) https://BioRender.com/byqbc3n, accessed on 26 April 2026.

Consistent with the progressive iron-retentive phenotype illustrated in Figure 1, human colorectal adenoma tissue studies have demonstrated increased expression of iron import proteins, including DMT1 and TfR1, together with functionally impaired iron export. Although FPN and the ferroxidase hephaestin are not always transcriptionally suppressed, their reduced expression and/or aberrant localization limit effective iron efflux, resulting in expansion of the intracellular labile iron pool. This shift supports proliferative metabolism while weakening cell adhesion, thereby favoring early neoplastic transformation [57].

As summarized in Figure 1, excess iron promotes oxidative stress through Fenton chemistry, leading to DNA damage and lipid peroxidation [56]. In line with this, elevated levels of 8-hydroxy-2′-deoxyguanosine (8-OHdG) have been documented in individuals with colorectal adenomas, accompanied by increased expression of base excision repair enzymes such as OGG1 and APE1 [58]. However, persistent oxidative lesions suggest that repair mechanisms do not fully compensate for ongoing reactive oxygen species generation. Lipid peroxidation products, including malondialdehyde (MDA) and 4-hydroxynonenal (4-HNE), are likewise increased and can actively promote proliferative and inflammatory signaling [59].

Mechanistically, increased iron availability intersects with oncogenic signaling pathways depicted in Figure 1. Experimental data from cell-based studies indicate that DMT1-mediated iron uptake can potentiate JAK–STAT3 activity, while restoration of APC function reduces intracellular iron through repression of TfR1 and DMT1. These observations support a functional, rather than purely associative, role for iron accumulation in early adenoma biology, though direct validation in human adenoma tissue remains limited [14].

Despite these mechanistic links, the relationship between circulating iron biomarkers and colorectal adenomas is notably heterogeneous, suggesting that subclinical iron dysregulation may be subtle and context-dependent. Among available markers, transferrin saturation (TSAT) has shown relatively consistent positive associations in some case–control settings. In one study including 100 adenoma cases and matched controls, higher TSAT was strongly associated with both overall and advanced adenomas (OR 3.05 and 2.71, respectively), even when ferritin was not informative, raising the possibility that circulating iron availability may better capture adenoma-related iron biology [45].

However, large prospective cohorts have not uniformly confirmed these findings. Within the Nurses’ Health Study and the PLCO screening trial, ferritin, serum iron, and TSAT were not significantly associated with adenoma risk, although inverse relationships were observed for total iron-binding capacity (TIBC) and unsaturated iron-binding capacity (UIBC). Interpretation is further complicated by the acute-phase behavior of ferritin and by the high prevalence of inflammation, obesity, and metabolic syndrome in colonoscopy screening populations. These inconsistencies likely reflect population heterogeneity, sex-specific effects, and variability in adenoma phenotype or timing of biomarker assessment. Notably, some analyses report stronger associations between iron indices and neoplasia risk in men, further underscoring the context-dependent nature of systemic iron markers [60,61].

Beyond traditional iron indices, emerging redox-related biomarkers may provide additional insight into the interplay between iron metabolism, obesity, and colorectal adenoma risk. High TSAT, together with reduced serum protein thiol (SHP) concentrations, reflecting systemic redox imbalance, has been proposed as a mechanistic link between iron overload and obesity-related adenoma risk. Consistently, SHP levels were inversely associated with advanced colorectal adenomas (OR 0.29; 95% CI 0.10–0.84), while ferritin concentrations correlated positively with red and processed meat intake and SHP levels were inversely associated with obesity, supporting a metabolic–redox interaction in early colorectal neoplasia [45].

At the tissue level, colorectal neoplastic cells demonstrate coordinated reprogramming of iron metabolism, including upregulation of TfR1, reduced ferroportin-mediated export, and dysregulated ferritin expression. IL-6-driven activation of hepcidin and STAT3 further reinforces intracellular iron retention and oxidative stress, promoting epithelial proliferation, immune evasion, and genomic instability. Given iron’s central role in cell-cycle control, mitochondrial function, and nucleotide metabolism, excess dietary iron, particularly heme iron, has been proposed as a contributor to colorectal carcinogenesis [13,16].

Iron dysregulation is best characterized in conventional adenomas arising through APC/Wnt pathway activation, where progressive upregulation of DMT1 and TfR1 has been documented along the normal mucosa–adenoma continuum, while APC loss further enhances intracellular iron accumulation. Experimental data indicate that luminal iron availability is a key modifier of intestinal tumorigenesis. In parallel, iron export becomes functionally impaired due to aberrant FPN localization and reduced hephaestin activity, promoting intracellular iron retention, increased proliferation, and reduced E-cadherin-mediated adhesion [57]. Excess iron also supports early metabolic reprogramming through glycolytic activation, ROS–NRF2 signaling, ferroptotic stress with adaptive resistance, and iron-dependent telomerase activation via Pirin [62,63]. By contrast, iron metabolism in serrated pathway lesions remains insufficiently defined. These polyps, characterized by BRAF or KRAS mutations and CpG island methylation, undergo marked epigenetic and metabolic reprogramming that may influence iron handling, but direct mechanistic data are still limited [64].

Collectively, the available evidence suggests that circulating iron biomarkers demonstrate heterogeneous performance for adenoma risk stratification, with TSAT showing more consistent associations than ferritin in some case–control settings, though results from large prospective cohorts remain discordant [45,60,61]. Emerging composite indices, such as the combination of high TSAT with reduced serum protein thiol concentrations reflecting systemic redox imbalance, may offer improved specificity for identifying individuals at elevated adenoma risk, particularly in the context of obesity and metabolic syndrome, but require prospective validation beyond their discovery cohort [45]. Beyond circulating indices, fecal transferrin testing has demonstrated higher positivity rates than immunochemical fecal occult blood testing for premalignant lesions, suggesting that iron-binding proteins in stool may capture adenoma-related biology that hemoglobin-based tests miss [65]. At the molecular imaging level, TfR1-targeted PET probes exploiting the iron-addicted phenotype of CRC cells have shown specific accumulation in TfR1-high colorectal tumors in preclinical models, positioning TfR1-directed imaging as a potential tool for noninvasive iron phenotyping and therapy stratification. Prospective validation of these approaches in well-defined adenoma cohorts, with stratification by lesion subtype and anatomical location, will be essential for establishing their clinical utility in CRC risk stratification [66,67]. The progressive alterations in iron metabolism across the normal mucosa–adenoma–carcinoma continuum are further summarized in Table 1.

## 6. Tumor Microenvironment and Iron Dysregulation

### 6.1. Iron Import Pathways in Colorectal Cancer Cells

**Figure 2:**
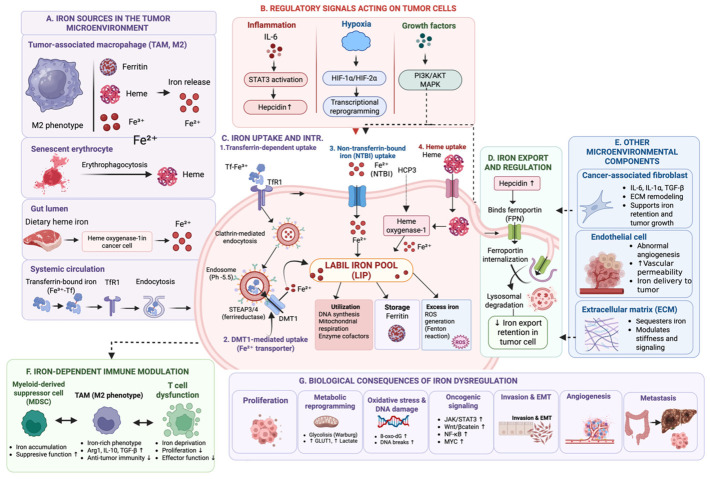
Integrated mechanisms of iron dysregulation in CRC cells and the tumor microenvironment. Iron sources including tumor-associated macrophages, senescent erythrocytes, dietary iron, and circulating transferrin-bound iron contribute to local iron availability (**A**). Inflammatory IL-6/STAT3, hypoxia-driven HIF signaling, and growth factor pathways promote intracellular iron retention (**B**). Iron uptake occurs via TfR1-mediated endocytosis, DMT1-dependent transport, NTBI uptake, and heme internalization, expanding the LIP (**C**). Simultaneously, hepcidin-induced FPN degradation suppresses iron export (**D**). CAFs, endothelial cells, and ECM interactions further shape iron distribution (**E**). Iron dysregulation promotes immunosuppressive phenotypes in MDSCs, TAMs, and T cells (**F**). Collectively, these processes drive oxidative stress, DNA damage, metabolic reprogramming, oncogenic signaling, angiogenesis, and metastatic dissemination (**G**). Solid arrows indicate iron flux and activation; dashed lines represent indirect interactions. Abbreviations: CRC, colorectal cancer; TfR1, transferrin receptor 1; DMT1, divalent metal transporter 1; DCYTB, duodenal cytochrome b; STEAP, six-transmembrane epithelial antigen of the prostate; NTBI, non-transferrin-bound iron; HCP1, heme carrier protein 1; HO-1, heme oxygenase-1; FPN, ferroportin; FT, ferritin; LIP, labile iron pool; ROS, reactive oxygen species; IL-6, interleukin-6; STAT3, signal transducer and activator of transcription 3; HIF, hypoxia-inducible factor; PI3K, phosphoinositide 3-kinase; AKT, protein kinase B; CAF, cancer-associated fibroblast; TAM, tumor-associated macrophage; ECM, extracellular matrix. Created in BioRender. Vladuta, T. (2026) https://BioRender.com/r4ivew9, accessed on 22 May 2026. Arrows indicate direction and magnitude of changes.

The tumor microenvironment in CRC supports a characteristic “iron addiction” phenotype, whereby tumor cells enhance iron acquisition and retention to sustain proliferation, metabolic plasticity, and survival. This state arises through coordinated upregulation of iron import pathways coupled with suppression of iron export mechanisms, as illustrated in Figure 2 [85]. Transferrin receptor 1 (TfR1/CD71), the principal mediator of transferrin-bound iron uptake, is consistently overexpressed in CRC and represents a central node in tumor iron metabolism. Multiple oncogenic pathways converge on TFRC regulation. The c-Myc transcription factor directly activates TFRC transcription, linking iron uptake to proliferative signaling, while hypoxia-inducible factors further enhance TfR1 and DMT1 expression under low-oxygen conditions. EGFR signaling also modulates TfR1 membrane localization and iron internalization, and more recently, IGF2BP2-mediated m6A modification of TFRC mRNA has emerged as an additional mechanism promoting iron accumulation in CRC cells [86]. Beyond its canonical role, nuclear TfR1 can interact with p53 and influence DNA damage–response programs, suggesting broader oncogenic functions [87].

Efficient iron uptake additionally depends on ferrireductase activity. Members of the STEAP family, particularly STEAP4, reduce Fe^3+^ to Fe^2+^ and facilitate cellular metal import [88]. STEAP4 is induced in inflammatory and hypoxic contexts and is upregulated in both inflammatory bowel disease and CRC, where it promotes mitochondrial iron loading, increases reactive oxygen species (ROS) generation, and enhances tumor burden in colitis-associated cancer models [74]. Recent work further implicates a STEAP4–NRF2–NQO1 signaling axis in supporting tumor cell survival and redox adaptation. In parallel with increased import, CRC cells develop robust iron-retention programs. Collectively, these mechanisms enhance iron acquisition and contribute to the expansion of the intracellular labile iron pool in CRC cells [73].

### 6.2. Iron Export Pathways in Colorectal Cancer Cells

Ferroportin (SLC40A1), the sole known cellular iron exporter, is frequently functionally suppressed in CRC, contributing to the iron-retentive phenotype of tumor cells. Although FPN transcripts may be preserved or even increased, the protein is often mislocalized intracellularly rather than at the plasma membrane, effectively blocking iron efflux. This defect is compounded by reduced expression of the ferroxidase hephaestin, resulting in expansion of the intracellular labile iron pool. Multiple regulatory layers contribute to ferroportin downregulation, including promoter methylation, microRNA-mediated repression (notably miR-194), and altered activity of transcriptional regulators such as NRF2 and MZF-1 [83]. Clinically, reduced FPN expression correlates with advanced disease, enhanced epithelial–mesenchymal transition, and poorer outcomes, whereas restoration of FPN function restrains tumor growth [57].

A central driver of iron sequestration in CRC is dysregulation of the hepcidin–ferroportin axis. Beyond hepatic production, CRC cells themselves express hepcidin, establishing an autocrine/paracrine loop that promotes ferroportin internalization and degradation. Tumor hepcidin expression is induced largely through IL-6/STAT3 signaling and increases with disease progression, particularly in metastatic settings. Functionally, hepcidin not only enhances tumor cell iron retention and proliferation but also contributes to immune suppression by promoting the accumulation of regulatory macrophages. Together, impaired iron export and enhanced hepcidin signaling establish the iron-retentive phenotype characteristic of CRC cells [89].

### 6.3. Intracellular Iron Storage and Retention

Intracellular iron homeostasis in CRC cells is governed by three interconnected regulatory axes, ferritin-mediated sequestration, NCOA4-dependent ferritinophagy, and IRP1/IRP2 post-transcriptional control, which are collectively reprogrammed in colorectal tumorigenesis to maintain an elevated labile iron pool (LIP) that sustains proliferative demands while attenuating oxidative cytotoxicity. Ferritin heavy chain 1 (FTH1), which harbors ferroxidase activity responsible for oxidizing Fe^2+^ to Fe^3+^ prior to mineral core deposition, is upregulated in CRC tumor tissue relative to adjacent normal mucosa, serving a dual function: limiting ROS-mediated cytotoxicity through Fe^2+^ sequestration while simultaneously maintaining a mobilizable iron reservoir for DNA synthesis and mitochondrial biogenesis [19,90].

Iron mobilization from ferritin stores is accomplished through NCOA4-mediated ferritinophagy, a selective autophagic pathway in which NCOA4 functions as a cargo receptor, binding directly to FTH1 subunits and targeting ferritin complexes to lysosomes for proteolytic degradation and Fe^2+^ liberation into the cytosolic LIP [91]. In CRC cells, activation of the NCOA4–FTH1 axis increases LIP and generates lipid ROS, contributing to ferroptotic pressure, while NCOA4 expression is regulated by transcription factors including TP53 and MYC [6,92].

Post-transcriptional regulation of iron storage is further mediated by IRP2, which is overexpressed in CRC tissues relative to normal mucosa and correlates with reduced overall survival [79,80]. Through stabilization of TfR1 mRNA and translational repression of ferritin, IRP2 simultaneously amplifies transferrin-mediated iron acquisition and attenuates storage capacity, establishing a sustained elevation of the intracellular LIP that confers a proliferative advantage to colorectal tumor cells [71,79].

### 6.4. Microenvironmental Iron Redistribution

Iron metabolism is tightly integrated with the tumor microenvironment. Iron-rich tumor-associated macrophages and M2 macrophage–derived ferritin can supply iron to cancer cells, while tumor-derived hepcidin further reinforces an immunosuppressive niche. IRP2 also contributes to iron dependency by stabilizing the expression of key iron-handling proteins and supporting tumor growth [93]. These findings establish iron import, retention, and redox adaptation as central drivers of CRC biology and highlight multiple therapeutic opportunities, including TfR1 targeting, hepcidin modulation, iron chelation, and ferroptosis induction [79,94].

The intratumoral microbiome represents an additional layer of iron regulation within the colorectal TME, actively hijacking local and systemic iron metabolism to promote tumor progression. Intratumoral bacteria, tumor cells, and host immune cells engage in tripartite competition for a finite iron pool, with pathogenic bacteria deploying siderophore-mediated iron acquisition systems that scavenge iron from the host environment [85,95]. *Fusobacterium nucleatum*, the best-characterized intratumoral pathobiont in CRC, amplifies tumor-promoting inflammation through a synergistic interaction with iron, whereby iron attenuates inhibitory NF-κB phosphorylation and amplifies *F. nucleatum*-induced CCL8 chemokine expression in macrophages, with elevated TSAT correlating with preferential iron deposition in TME macrophages [96]. *Peptostreptococcus anaerobius* produces the tryptophan metabolite trans-3-indoleacrylic acid, which activates the AHR–ALDH1A3–FSP1–CoQ axis to suppress lipid peroxidation and confer ferroptosis resistance to CRC cells [97,98]. Furthermore, tumor-associated macrophages secrete iron-loaded lipocalin-2 as a transferrin-independent iron delivery system to tumor cells, sustaining proliferation and chemoresistance [99].

### 6.5. Downstream Biological Consequences of Iron Dysregulation

Net iron accumulation in CRC reflects the combined effects of impaired export and increased import via DMT1, TfR1, and DCYTB, with HIF-2α acting as a key transcriptional driver of DMT1 [100].

Elevated intracellular iron drives several oncogenic programs. Iron promotes glycolytic reprogramming through GLUT1 upregulation and PDH inhibition, supporting the Warburg phenotype, and ROS generated by excess iron activates NRF2 signaling while simultaneously creating ferroptotic pressure [12,62]. Nevertheless, CRC cells frequently acquire adaptive resistance via upregulation of SLC7A11 and GPX4 [62]. Iron also interfaces with proliferative signaling through a DMT1–JAK–STAT3 axis and can enhance telomerase activity via the iron sensor Pirin, whereas heme oxygenase-1 provides an additional context-dependent source of redox-active iron during inflammation [84]. Collectively, these mechanisms establish iron import and retention as central drivers of colorectal tumor biology [89].

A further downstream consequence of iron dysregulation in CRC is the phenomenon of porphyrin overdrive, a cancer-specific rewiring of heme biosynthesis in which ferrochelatase (FECH), the terminal enzyme responsible for inserting Fe^2+^ into protoporphyrin IX (PpIX), is consistently downregulated across multiple CRC datasets, while mid-pathway enzymes remain active, resulting in endogenous PpIX accumulation within tumor cells [101,102]. This accumulation has been detected naturally in primary CRC tissues and lymph node metastases in the absence of exogenous ALA administration [103]. Porphyrin overdrive has been proposed to reflect an iron-addicted, early embryonic-like stem metabolic state that supports redox adaptation while creating vulnerabilities to iron–heme pathway targeting; however, this hypothesis remains conceptually compelling rather than experimentally validated in CRC, with the strongest supporting evidence deriving from iron metabolism gene signatures in cancer stem cells and from the heme–SDH–CoQ axis through which CRC cells co-opt heme-dependent succinate dehydrogenase activity to buffer iron-induced oxidative stress [104].

### 6.6. Mitochondrial Iron Metabolism

**Figure 3:**
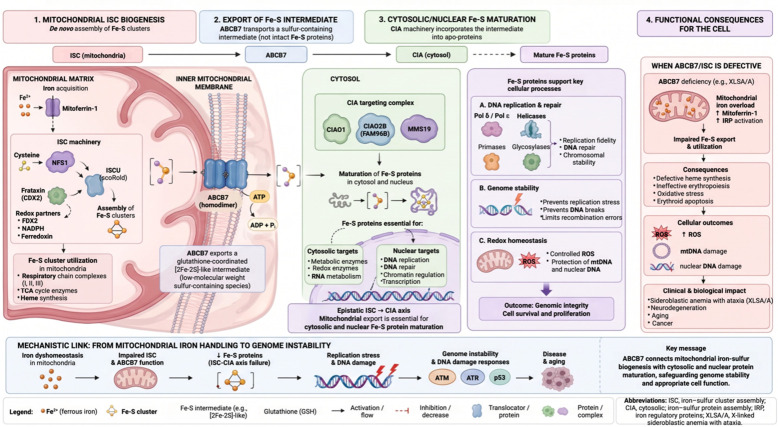
Mitochondrial iron–sulfur cluster biogenesis and the ABCB7–ISC–CIA axis linking mitochondrial iron metabolism to genome stability. Fe–S clusters are assembled de novo on the ISCU scaffold by the ISC machinery (NFS1, frataxin) within the mitochondrial matrix (step 1). ABCB7 exports a glutathione-coordinated Fe–S intermediate to the cytosol (step 2), where the CIA targeting complex (CIAO1, CIAO2B/FAM96B, MMS19) matures cytosolic and nuclear Fe–S proteins required for DNA replication, repair, and chromatin regulation (steps 3–4). Disruption of ISC components or ABCB7 impairs Fe–S biogenesis, causing mitochondrial iron accumulation, defective heme synthesis, replication stress, genomic instability, and activation of DNA damage responses, establishing a mechanistic link between mitochondrial iron handling and nuclear genome integrity relevant to cancer progression. Abbreviations: ISC, iron–sulfur cluster assembly; CIA, cytosolic iron–sulfur protein assembly; Fe–S, iron–sulfur cluster; ABCB7, ATP-binding cassette subfamily B member 7; ISCU, iron–sulfur cluster scaffold protein; NFS1, cysteine desulfurase; FDX2, ferredoxin 2; NADPH, nicotinamide adenine dinucleotide phosphate (reduced form); CIAO1, cytosolic iron–sulfur assembly component 1; CIAO2B (FAM96B), cytosolic iron–sulfur assembly component 2B; MMS19, MMS19 homolog; ATP, adenosine triphosphate; ADP, adenosine diphosphate; Pi, inorganic phosphate; mtDNA, mitochondrial DNA; ROS, reactive oxygen species; IRP, iron-regulatory proteins; ATM, ataxia telangiectasia mutated; ATR, ataxia telangiectasia and Rad3-related; p53, tumor protein p53; TCA cycle, tricarboxylic acid cycle; XLSA/A, X-linked sideroblastic anemia with ataxia; GSH, glutathione. Created in BioRender. Vladuta, T. (2026) https://BioRender.com/o1edbwr, accessed on 26 April 2026. Arrows indicate direction and magnitude of changes.

Mitochondria represent the central hub of cellular iron metabolism, coordinating the biosynthesis of iron–sulfur (Fe–S) clusters and heme—two essential cofactors required for respiration, metabolism, and genome maintenance. As illustrated in Figure 3, a key component of this system is ABCB7, an ATP-binding cassette transporter located in the inner mitochondrial membrane that mediates export of Fe–S cluster-related intermediates from mitochondria to the cytosol. ABCB7 is evolutionarily conserved from yeast Atm1 and exports glutathione-coordinated [2Fe–2S] intermediates generated by the ISC machinery (NFS1, frataxin, ISCU) to the cytosol, where the CIA targeting complex (CIAO1, CIAO2B/FAM96B, MMS19) matures Fe–S proteins required for DNA replication, repair, and chromosomal stability, establishing an epistatic ISC → CIA axis that links mitochondrial iron handling to nuclear genome integrity [105,106,107,108]. Disruption of either ISC components or ABCB7 results in combined mitochondrial and cytosolic Fe–S deficiency, promoting mitochondrial iron accumulation, defective heme synthesis, and increased oxidative stress [105].

Many DNA metabolic enzymes depend critically on Fe–S cofactors for their structural integrity and catalytic activity. Replicative DNA polymerases δ and ε, DNA helicases, primases, and glycosylases all contain redox-active [4Fe–4S] clusters, such that impaired Fe–S biogenesis leads directly to replication stress, defective DNA repair, increased recombination, and activation of DNA damage responses. Experimental models confirm that reduced Fe–S availability, rather than loss of respiration alone, is sufficient to trigger nuclear genome instability [109].

Although dedicated expression profiling studies have not yet consistently identified ABCB7 as differentially expressed in CRC tissues, converging evidence implicates the broader ISC–CIA axis in colorectal tumorigenesis through several CRC-specific mechanisms [110,111]. Loss of p53 function, occurring in approximately 79% of CRC cases, disrupts the p53–ISCU regulatory axis, impairing Fe–S cluster assembly and driving compensatory iron accumulation and oxidative stress in tumor cells [112,113]. Furthermore, MUTYH, a base excision repair glycosylase requiring an Fe–S cluster for catalytic function, represents a direct mechanistic link between CIA pathway dysfunction and colorectal adenoma formation, as biallelic MUTYH inactivation causes MUTYH-associated polyposis [114,115]. Dysregulation of the CIA pathway flux through MAGE-F1-mediated degradation of MMS19 has been identified as a recurrent feature of human cancers associated with increased mutational burden, further implicating impaired cytosolic Fe–S protein maturation in CRC genomic instability [116]. Additionally, the Fe–S cluster assembly factor FDX2 has been shown to be specifically required for tumor initiation and metastasis under hypoxic conditions, a prominent feature of the colorectal tumor microenvironment, suggesting a stage-specific dependency on mitochondrial iron–sulfur biogenesis during CRC progression [117].

In parallel, mitochondrial iron dysregulation enhances reactive oxygen species production, damaging both mitochondrial DNA and nuclear DNA and reinforcing a feed-forward cycle of mitochondrial dysfunction and genomic injury, as illustrated in Figure 3. Together, the ABCB7-ISC-CIA axis represents a critical mechanistic bridge between mitochondrial iron metabolism and genome stability, with important implications for hematologic disease and cancer progression, including CRC [114].

## 7. Iron-Induced Oxidative Stress and Molecular Damage

**Figure 4:**
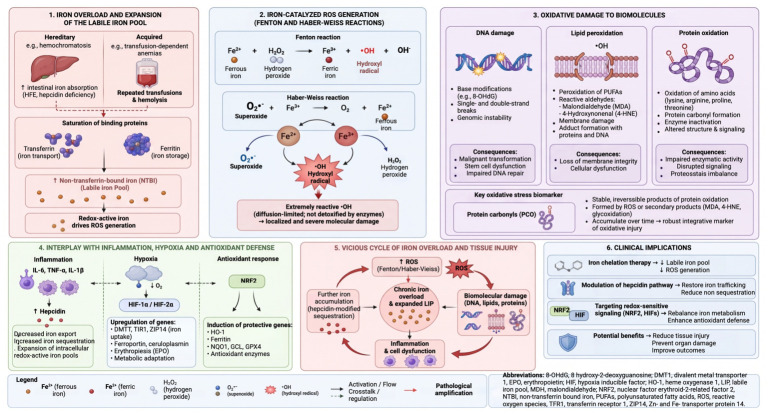
Mechanistic overview of iron-induced oxidative stress and biomolecular damage. Iron excess saturates transferrin and ferritin buffering capacity, leading to NTBI accumulation and LIP expansion (1). Redox-active iron drives Fenton and Haber–Weiss reactions, generating hydroxyl radicals (2). These species induce oxidative damage to DNA (8-OHdG, strand breaks), lipids (MDA, 4-HNE), and proteins (PCO), with protein carbonyls serving as stable cumulative injury markers (3). Iron toxicity is amplified by inflammatory signaling, hepcidin-mediated iron sequestration, and hypoxia-driven pathway activation, partially counterbalanced by NRF2-dependent antioxidant defense (4). Together, these processes establish a self-sustaining cycle of ROS generation and progressive biomolecular damage (5) with implications for iron chelation, iron-regulatory pathway modulation, and redox-targeted therapeutic strategies (6). Abbreviations: 8-OHdG, 8-hydroxy-2′-deoxyguanosine; DMT1, divalent metal transporter 1; EPO, erythropoietin; Fe^2+^, ferrous iron; Fe^3+^, ferric iron; H_2_O_2_, hydrogen peroxide; HIF, hypoxia-inducible factor; HO-1, heme oxygenase-1; IL-1β, interleukin-1 beta; LIP, labile iron pool; MDA, malondialdehyde; NRF2, nuclear factor erythroid 2–related factor 2; NTBI, non-transferrin-bound iron; O_2_•^−^, superoxide anion; •OH, hydroxyl radical; PCO, protein carbonyls; PUFA, polyunsaturated fatty acids; ROS, reactive oxygen species; TfR1, transferrin receptor 1; 4-HNE, 4-hydroxynonenal. Created in BioRender. Vladuta, T. (2026) https://BioRender.com/yb58bjj, accessed on 11 June 2026. Arrows indicate direction and magnitude of changes.

Iron is an essential transition metal required for oxygen transport, mitochondrial respiration, DNA synthesis, and cellular metabolism. However, the same redox properties that make iron indispensable also render it potentially toxic when present in excess. Iron overload, whether hereditary (e.g., hemochromatosis) or acquired (e.g., transfusion-dependent anemias), leads to progressive tissue injury primarily through oxidative stress-mediated mechanisms. Central to this process, as illustrated in Figure 4, Panel 1, is the expansion of the labile iron pool, which fuels the generation of reactive oxygen species. In CRC, expansion of the labile iron pool has been documented both in preneoplastic adenomas and in established carcinomas, where it drives a progressive intensification of oxidative stress along the adenoma–carcinoma sequence [93].

The biochemical basis of iron toxicity lies in the Fenton and Haber–Weiss reactions (Figure 4, panel 2). In the Fenton reaction, ferrous iron (Fe^2+^) reacts with hydrogen peroxide (H_2_O_2_) to generate the highly reactive hydroxyl radical (•OH). Ferric iron (Fe^3+^) can then be reduced back to Fe^2+^ by superoxide (O_2_•^−^), sustaining a catalytic redox cycle. Because hydroxyl radicals react at diffusion-limited rates with nearby biomolecules and cannot be enzymatically detoxified, their formation results in highly localized and severe molecular damage. Under physiological conditions, iron is safely sequestered by ferritin and transferrin; however, in iron overload states, these buffering systems become saturated, leading to the appearance of non-transferrin-bound iron (NTBI), a highly redox-active form that readily participates in ROS generation [118].

Iron-catalyzed oxidative stress targets all major classes of biomolecules (Figure 4, panel 3). DNA damage includes base modifications such as 8-OHdG, single- and double-strand breaks, and genomic instability, which may contribute to malignant transformation and stem cell dysfunction. In CRC, 8-OHdG levels are significantly elevated in tumor tissue compared to adjacent normal mucosa, with approximately twofold higher concentrations documented in carcinoma tissue, while systemic oxidative DNA damage, assessed by 8-OHdG in leukocytes and urine, is already detectable at the adenoma stage [58,119]. Lipid peroxidation of polyunsaturated fatty acids generates reactive aldehydes, particularly malondialdehyde (MDA) and 4-hydroxynonenal (4-HNE), which disrupt membrane integrity and form adducts with proteins and nucleic acids. In CRC tissue, MDA concentrations are approximately twofold higher than in normal colon, with the highest levels observed in mucinous adenocarcinomas and stage IV disease, while 4-HNE is selectively cytotoxic to normal colonocytes but not to APC-mutant preneoplastic cells, creating a selection pressure that promotes expansion of premalignant clones [120,121,122]. Protein oxidation is another major consequence, leading to enzyme inactivation, structural alterations, and impaired cellular signaling [120,123].

Among oxidative stress biomarkers, protein carbonyls (PCO) are especially valuable because they represent stable, irreversible products of protein oxidation. (Figure 4, panel 3) Carbonyl groups arise either from direct ROS-mediated oxidation of susceptible amino acids (lysine, arginine, proline, threonine) or from secondary modification by lipid peroxidation products and glycoxidation reactions. Because PCOs accumulate over time and are relatively resistant to further metabolism, they serve as robust integrative markers of oxidative injury in iron overload and other chronic diseases [124]. In CRC patients, PCO levels are significantly elevated compared to healthy controls, while 3-nitrotyrosine, a marker of nitrosative protein damage, is detected in carcinoma cells and correlates with proliferative activity, further linking oxidative and nitrosative stress to tumor progression [125].

Iron toxicity is amplified by its interaction with inflammation and hypoxia (Figure 4, panel 4). Inflammatory cytokines, particularly IL-6, induce hepcidin, altering iron trafficking and promoting intracellular iron sequestration, which can expand redox-active pools locally. Concurrently, hypoxia-inducible factors (HIFs) tightly link oxygen sensing to iron metabolism by regulating genes involved in iron absorption, transport, and erythropoiesis. The antioxidant transcription factor NRF2 provides a counter-regulatory mechanism by inducing detoxifying and iron-handling proteins [126]. Within the colorectal tumor microenvironment, NADPH oxidase 1 (NOX1), the dominant superoxide-generating enzyme in colonic epithelium, is consistently overexpressed in both adenomas and well-differentiated adenocarcinomas, and its expression is directly upregulated by KRAS mutations, linking the most prevalent CRC driver mutation to ROS generation. Pro-inflammatory cytokines IL-4 and IL-13 further amplify NOX1 expression via JAK1/STAT6/GATA3 signaling, establishing a feed-forward loop between inflammation and oxidative stress in the TME [127,128].

Disruption of the balance among iron overload, inflammatory signaling, and hypoxic adaptation creates a self-reinforcing cycle of ROS generation and tissue injury (Figure 4, panel 5). In colorectal carcinogenesis, this cycle is further amplified by heme iron-driven luminal lipid peroxidation, which generates cytotoxic aldehydes that selectively eliminate normal colonocytes while sparing APC-mutant preneoplastic cells through NRF2-dependent antioxidant adaptation, thereby creating a pro-tumorigenic selection environment in the colonic lumen [121,122]. Understanding this integrated network has important implications for therapeutic strategies, including iron chelation, modulation of hepcidin pathways, and targeting of redox-sensitive signaling systems [123].

## 8. Ferroptosis as an Iron-Dependent Therapeutic Vulnerability in Colorectal Cancer

Ferroptosis is a form of regulated cell death mechanistically distinct from apoptosis and necrosis, driven by iron-catalyzed accumulation of lipid peroxides to lethal levels. In CRC, the iron-addicted phenotype that sustains tumor proliferation simultaneously positions malignant cells close to the ferroptotic threshold, creating a paradoxical vulnerability that can be exploited to overcome conventional chemotherapy resistance [129,130].

### 8.1. The Ferroptosis Regulatory Network in Colorectal Cancer

Ferroptosis execution depends on three converging processes: iron-catalyzed generation of reactive hydroxyl radicals via Fenton chemistry; oxidation of polyunsaturated fatty acid-containing phospholipids mediated by ACSL4/LPCAT3 and lipoxygenases; and failure of the antioxidant defense system, primarily GPX4, which neutralizes lipid peroxides using glutathione as a cofactor [131].

CRC cells maintain multiple defense systems against ferroptosis. The most extensively studied is the system Xc^−^/GSH/GPX4 axis, in which SLC7A11 imports cystine for glutathione synthesis, sustaining GPX4-mediated lipid peroxide detoxification. SLC7A11 expression is regulated by multiple factors, including transcriptional activation by LEF1 and post-transcriptional stabilization through PRMT5 and USP5, while RBMS2 promotes mRNA destabilization and acts as a pro-ferroptotic tumor suppressor [132,133,134,135]. A parallel defense is provided by ferroptosis suppressor protein 1 (FSP1), which reduces coenzyme Q10 at the plasma membrane, acting as a GPX4-independent antioxidant that is upregulated in KRAS-mutant CRC cells [136,137].

NRF2 serves as a central transcriptional regulator of ferroptosis sensitivity in CRC, activating SLC7A11, GPX4, FTH1, and FPN, while also inducing HMOX1, which releases iron from heme catabolism and exerts context-dependent pro-ferroptotic effects [83,138]. A recently identified buffering mechanism, the heme–SDH–CoQ axis, enables CRC cells to tolerate high intracellular iron by channeling it into heme, driving SDH-dependent reduction of coenzyme Q to ubiquinol, which acts as a membrane-localized lipid radical trap. This mechanism allows CRC cells to exploit the same metabolic cofactors for both energy production and ferroptosis resistance, and its disruption represents a therapeutic vulnerability distinct from GPX4 or system Xc^−^ targeting [104].

### 8.2. Exploiting Iron Addiction to Overcome Chemotherapy Resistance

The elevated intracellular iron load in CRC narrows the margin between productive iron utilization and oxidative toxicity, rendering iron-addicted tumor cells inherently more susceptible to ferroptosis induction than normal colonocytes. This vulnerability is especially pronounced in persister cancer cells, dedifferentiated, therapy-resistant subpopulations that survive conventional chemotherapy and accumulate PUFA-enriched phospholipids, making ferroptosis their principal therapeutic liability [139].

Chemoresistant CRC cells suppress ferroptosis through several mechanisms: NRF2/GPX4 upregulation in oxaliplatin-resistant cells, ferritin-mediated iron sequestration, SREBP1-driven accumulation of monounsaturated fatty acids that displace ferroptosis-susceptible PUFAs from membrane phospholipids, and FOSL1-mediated transcriptional activation of SRSF2 [140,141,142]. The FBXL5–IRP2–TfR1 axis provides an additional resistance mechanism, whereby FBXL5 depletion increases IRP2 and TfR1 expression, expands the labile iron pool, and restores ferroptosis sensitivity in oxaliplatin-resistant cells [143].

Several combination strategies have demonstrated preclinical efficacy. System Xc^−^ inhibition with erastin synergizes with oxaliplatin in resistant CRC cells. KRAS G12D inhibition with MRTX-1133 potentiates ferroptosis induction in patient-derived organoids. Sodium butyrate selectively induces ferroptosis in CRC cells through NCOA4-mediated ferritinophagy and labile iron pool expansion without toxicity to normal colonocytes [130,131,136]. At the immunological level, IFN-γ secreted by CD8^+^ T cells suppresses SLC7A11 in tumor cells, reducing glutathione availability and sensitizing CRC to ferroptosis, creating a positive feedback loop between immune activation and ferroptotic cell death [129,144].

### 8.3. Cell Death Modality Balance and Therapy–Iron Interactions in the Tumor Microenvironment

The functional role of iron within the colorectal TME is ultimately governed by the balance among apoptosis, necrosis, and ferroptosis, which determines whether iron acts as a trophic factor supporting tumor growth, an inflammatory amplifier reshaping immune contexture, or a therapeutic vulnerability [129,145]. When antioxidant defenses are intact, iron sustains DNA synthesis, mitochondrial respiration, and iron–sulfur cluster-dependent enzyme activity. When ferroptosis proceeds in a spatially restricted manner, dying cells release damage-associated molecular patterns and pro-inflammatory mediators that activate dendritic cells and cytotoxic T lymphocytes, a process termed immunogenic ferroptosis, potentially converting immunologically cold tumors into hot tumors responsive to immune checkpoint blockade [129,144].

Ferroptosis exhibits functional crosstalk with other cell death pathways. Iron–sulfur cluster disruption can activate the intrinsic apoptotic cascade, while NRF2 suppression co-activates ferroptosis and GSDME-mediated pyroptosis [112,141]. Phosphorylated NFS1 participates in PANoptosome assembly, linking iron–sulfur cluster homeostasis to the simultaneous activation of pyroptosis, apoptosis, and necroptosis [112]. Importantly, CRC cells that have acquired apoptosis resistance frequently retain ferroptosis sensitivity, as lipid peroxidation-mediated execution is mechanistically independent of the apoptotic caspase machinery, providing the rationale for ferroptosis-based approaches in chemoresistant disease [146,147].

Conventional therapies also exert bidirectional effects on iron homeostasis that modulate ferroptosis susceptibility. Oxaliplatin activates NRF2, simultaneously upregulating GPX4 and HMOX1, with opposing ferroptotic consequences whose net outcome depends on their relative magnitude [138,141]. Ionizing radiation generates ROS that synergize with Fenton chemistry to amplify lipid peroxidation while inducing adaptive changes in iron-regulatory gene expression. These bidirectional therapy–iron interactions highlight the importance of incorporating ferroptosis vulnerability into the design of combination therapeutic strategies for CRC [137].

The therapeutic strategies discussed above are summarized in Table 2, which provides an overview of current and emerging approaches targeting iron metabolism in CRC.

## 9. Current Challenges and Future Perspectives

Future progress in the field of iron metabolism in colorectal cancer will depend on improving methodological consistency, refining biological resolution, and strengthening the link between mechanistic insights and clinically relevant data. One of the major challenges remains the heterogeneity of findings across epidemiological and experimental studies, particularly regarding the role of dietary iron. Differences in study design, variability in exposure assessment, and the influence of confounding factors such as inflammation, metabolic status, and gut microbiota contribute to inconsistent conclusions. In addition, widely used systemic iron biomarkers, including ferritin and transferrin saturation, are strongly affected by acute-phase responses and may not accurately reflect iron availability at the tissue or tumor level. These limitations continue to complicate the interpretation of iron-related risk and highlight the need for more standardized and context-specific analytical approaches.

Another important challenge lies in the limited understanding of iron distribution and function within the tumor microenvironment. Most current data rely on bulk analyses or preclinical models, which do not capture the spatial and cellular heterogeneity of colorectal tumors. As a result, the dynamic interactions between tumor cells, immune populations, and stromal components in regulating iron availability remain incompletely defined. This issue is particularly relevant for early tumorigenesis, where local iron accumulation appears to play a critical role, yet remains difficult to quantify and characterize. Furthermore, while iron dysregulation has been extensively studied in conventional adenomas, its role in serrated pathway lesions is still poorly understood, representing a significant gap in current knowledge.

From a translational perspective, several iron metabolism biomarkers have demonstrated promising clinical relevance in CRC. Serum hepcidin has emerged as a prognostically relevant biomarker, with baseline levels above 40 ng/mL independently associated with significantly shorter overall survival in metastatic CRC patients receiving first-line chemotherapy, remaining significant after adjustment for CEA, performance status, and RAS/BRAF mutation status [156]. Additionally, iron deficiency affects approximately 50% of CRC patients at diagnosis and is independently associated with larger tumor diameter, more advanced stage, poorer differentiation, and reduced response to neoadjuvant chemoradiotherapy, underscoring the clinical importance of perioperative iron assessment and correction [157,158]. At the molecular level, ferroptosis-related gene signatures, including a composite score incorporating GPX4, NOX1, and FACL4, have shown clinical validation in predicting adjuvant chemotherapy benefit in stage II/III CRC, with low-score patients deriving significant progression-free and overall survival benefit from adjuvant chemotherapy while high-score patients showed no statistically significant benefit, suggesting potential utility for treatment stratification [159].

Therapeutic strategies targeting iron metabolism are gaining increasing attention but face important translational barriers. A fundamental challenge is the iron chelation–ferroptosis paradox: iron chelation depletes tumor iron to inhibit proliferation, while ferroptosis induction requires intracellular iron to execute oxidative cell death, two opposing strategies whose optimal application depends on tumor iron status, antioxidant capacity, and disease stage [19,160]. Several FDA-approved drugs with repurposing potential, including deferiprone, CX-5461, auranofin, sulfasalazine, and sorafenib, have demonstrated preclinical efficacy in CRC models, while first-in-class agents such as acevaltrate have outperformed standard CRC drugs in animal models and patient-derived organoids [19,161]. The RSL3 + HIF-1α inhibitor + anti-PD-1 triple combination has shown particular promise in converting immunologically cold MSS CRC, representing over 80% of cases, into hot tumors with enhanced CD8^+^ T cell infiltration, addressing a critical unmet need in checkpoint inhibitor resistance [162]. Future biomarker-guided clinical trials should incorporate baseline serum hepcidin and tissue iron metabolism profiling for patient stratification, ferroptosis score assessment for predicting chemotherapy responsiveness, and molecular subtyping by MSI status and KRAS/BRAF mutation to match patients with appropriate iron-targeting strategies [16,130].

Taken together, advancing this field will require a shift from descriptive and often fragmented observations toward integrated and mechanistically grounded models of iron metabolism in CRC. Rather than focusing on single biomarkers, emerging approaches combining multi-omics data, spatial profiling, and single-cell resolution will be essential for capturing the cellular heterogeneity of iron-dependent processes within the colorectal TME and for translating current mechanistic insights into reliable biomarkers and effective therapeutic strategies.

## 10. Conclusions

Iron metabolism represents a key regulatory axis in colorectal tumorigenesis, contributing to the transition from preneoplastic lesions to invasive cancer through coordinated effects on cellular proliferation, redox homeostasis, and metabolic reprogramming. Alterations in iron handling, including increased iron uptake, impaired ferroportin-mediated export, and expansion of the labile iron pool, are evident early in colorectal neoplasia and progressively integrate into tumor biology, supporting DNA synthesis, promoting oxidative stress-induced genomic instability, and activating oncogenic signaling pathways while also shaping the tumor microenvironment through interactions with immune and stromal components.

The role of iron in colorectal cancer is highly context-dependent, reflecting the complex interplay between luminal exposure, systemic iron status, and local tumor iron retention. The apparent coexistence of iron deficiency at the systemic level with iron accumulation within tumor cells underscores the need to distinguish between these compartments when interpreting clinical and experimental data. Epidemiological evidence linking dietary iron to CRC risk remains heterogeneous and should be interpreted with appropriate caution, given the difficulty of disentangling luminal from systemic iron effects, the confounding influence of inflammatory and metabolic comorbidities on circulating biomarkers, and the potential for reverse causation.

Several important knowledge gaps remain. Iron metabolism in serrated pathway lesions, characterized by BRAF or KRAS mutations and CpG island methylation, is insufficiently characterized compared to conventional adenomas, representing a significant gap given the growing clinical relevance of this pathway. Furthermore, most mechanistic evidence derives from bulk tissue analyses or preclinical models that do not capture the spatial and cellular heterogeneity of the colorectal tumor microenvironment; spatially resolved and cell-specific studies of iron distribution across tumor, stromal, and immune compartments will be essential for a more complete understanding of iron-dependent tumor–microenvironment interactions. Tissue-level iron biomarkers, including iron-regulatory protein expression profiles and oxidative damage signatures, require prospective validation in clinically well-defined CRC cohorts before their utility for risk stratification or therapeutic guidance can be established.

The translational relevance of iron metabolism as a source of biomarkers and therapeutic targets is promising but should be presented with appropriate caution. Strategies aimed at modulating iron availability, including hepcidin–ferroportin axis modulation, iron chelation, and targeted induction of ferroptosis, have demonstrated preclinical efficacy but still require rigorous validation in CRC patients. Integrating iron metabolism into multidimensional models of CRC biology, combining spatial profiling, multi-omics approaches, and clinically annotated cohort data, will be essential for translating current mechanistic insights into reliable biomarkers and effective therapeutic strategies.

## Figures and Tables

**Table 1:** Progressive remodeling of iron metabolism across the colorectal tumorigenesis continuum.

Parameter	Normal Colonic Mucosa	Colorectal Adenoma	Colorectal Carcinoma	References
**Systemic Iron Biomarkers**				
**Serum iron**	Approximately 108 µg/dL under physiological conditions	Reduced concentrations reported in adenomas ≥ 1 cm, potentially reflecting occult blood loss	Mean levels around 54.5 µg/dL despite substantial intratumoral iron accumulation	[68,69]
**Serum ferritin**	Physiological range approximately 102 µg/L	Variable findings; lower concentrations in bleeding adenomas, whereas higher ferritin levels have been associated with adenoma risk in some studies; meta-analysis shows null association overall	Lower than controls (~60.4 µg/L); concentrations exceeding 100 µg/L associated with approximately fivefold elevated risk of advanced colonic neoplasia in some cohorts; overall findings remain discordant	[20,69]
**TSAT**	Within physiological range	Positively associated with overall and advanced adenoma risk (OR 3.05 and 2.71, respectively)	Associations remain inconsistent across CRC cohorts	[45,60]
**TIBC/UIBC**	Physiological values	Lower TIBC associated with reduced adenoma risk	Limited evidence available	[60]
**Iron Import**				
**TfR1**	Low basal expression, predominantly in proliferative crypt cells	Increased expression detected during the normal mucosa–adenoma transition	Marked overexpression in well-differentiated and early-stage tumors (Dukes A/B); paradoxical decline in poorly differentiated and metastatic disease (Dukes C/D); overexpression correlates with worse overall survival, higher tumor mutational burden, and upregulated immune checkpoints	[16,70]
**DMT1**	Low constitutive expression	Not well characterized in adenoma tissue	Markedly elevated expression supporting enhanced iron uptake	[14,71]
**DCYTB**	Minimal basal expression in colonic epithelium; primarily expressed in duodenum	Insufficient evidence available	Elevated at both transcript and protein levels; co-upregulated with DMT1 and TfR1; facilitates reduction of ferric to ferrous iron for cellular import	[57,72]
**STEAP4**	Low physiological expression	Not characterized	Upregulated under inflammatory and hypoxic conditions; associated with enhanced intracellular iron loading	[73,74]
**HIF-2α**	Minimal activity under normoxic conditions	Not characterized	Activated in hypoxic tumor regions; promotes transcription of iron import genes	[75]
**Iron Export**				
**FPN (Ferroportin)**	Membrane-localized and functionally active	Not well characterized in adenoma tissue	Frequently mislocalized intracellularly, resulting in impaired iron efflux	[16]
**Hephaestin**	Normal basolateral expression	Reduced activity observed in adenomatous tissue	Downregulated expression further limits cellular iron export	[16]
**Hepcidin**	Minimal local expression	Not sufficiently investigated	Markedly elevated local production promoting ferroportin degradation and iron retention	[76,77]
**Iron Storage and Regulation**				
**FTH1/Ferritin**	Low expression within crypt-base epithelial cells	Detectable mainly in dysplastic lesions	Increased expression supporting iron sequestration and protection against oxidative stress	[49,68]
**NCOA4**	Physiological ferritin turnover and iron mobilization	Not characterized	Enhanced ferritinophagy contributing to labile iron pool expansion and ROS generation	[78]
**IRP2**	Low basal activity	Not characterized	Frequently overexpressed; associated with increased TfR1 expression, BRAF mutations, and unfavorable outcomes	[79,80]
**Iron Transport in TME**				
**NGAL/LCN2**	Weak or absent epithelial expression	Expressed in adenomatous epithelium; expression increases with adenoma size	Elevated in CRC; suppresses ferroptosis by inducing GPX4 and SLC7A11; promotes chemoresistance	[81]
**Downstream Biological Consequences**				
**NRF2**	Basal antioxidant and cytoprotective activity	Reduced expression reported in sporadic adenomas	Activated signaling promotes antioxidant adaptation and ferroptosis resistance	[82]
**SLC7A11/GPX4**	Physiological antioxidant defense	Not characterized	Upregulated as part of an adaptive response to iron-induced oxidative stress	[62,83]
**HO-1**	Low basal expression; inducible during inflammation	Not characterized	Elevated expression increases heme degradation and iron-dependent redox signaling	[84]
**miR-194**	Physiological expression levels	Iron-related miRNA alterations detectable during early neoplastic stages	Contributes to ferroportin repression and impaired iron export	[71]
**E-cadherin**	Preserved epithelial membrane expression	Not specifically evaluated in the context of iron metabolism	Reduced expression associated with iron-driven epithelial–mesenchymal transition	[57]
**Net Functional Effect**				
**Overall iron status**	Balanced iron import and export	Early iron-related molecular alterations detectable; systemic iron indices variably associated with neoplasia risk	Iron import maximized (↑ TfR1, ↑ DMT1, ↑ DCYTB, ↑ STEAP4) + iron export blocked (↓ FPN, ↓ HEPH, ↑ hepcidin) + iron regulation reprogrammed (↑ IRP2, ↑ HIF-2α, ↑ NRF2) → LIP expansion → proliferation, EMT, Warburg effect, and ferroptosis resistance	[19,57,83]

Arrows are used in their conventional sense to indicate increase (↑) or decrease (↓).

**Table 2:** Current and emerging therapeutic strategies targeting iron metabolism in colorectal cancer.

Category	Agent/Approach	Mechanism of Action	Key CRC Evidence	Development Stage	References
**Iron Chelators**					
	Deferoxamine (DFO)	Depletes intracellular labile iron pool through Fe(III) chelation	Inhibits multiple CRC cell lines; reduces CDK1/POLD1 in patient-derived colonoids; stabilizes p53	Preclinical in CRC; Phase I/II in other malignancies	[19]
	Deferasirox (DFX)	Oral Fe(III) chelator; depletes cellular iron and upregulates NDRG1	Antiproliferative activity in gastrointestinal cancer cell lines; novel derivatives show selective cytotoxicity in metastatic CRC	Preclinical; oral bioavailability advantageous over DFO	[148]
**TfR1 Targeting**					
	Anti-TfR1 antibodies	Disrupts transferrin-mediated iron uptake; Fc-mediated immune effector functions	TfR1 overexpression in CRC correlates with worse overall survival and upregulated immune checkpoints; high TFRC associated with inflamed TME	Preclinical and early clinical in other cancers	[149]
	^68^Ga-labeled PET probes	Noninvasive PET imaging of TfR1 expression for patient stratification	Specific accumulation in TfR1-high CRC xenografts; tumor-to-muscle ratio 7.88; signal reduction > 83% with blocking	Preclinical (2025)	[67]
**Hepcidin Modulation**					
	Anti-hepcidin antibodies (LY2787106)	Neutralizes circulating hepcidin; restores FPN membrane expression and iron efflux from tumor cells	CRC cells produce autocrine hepcidin; hepcidin silencing reduces EMT markers and regulatory macrophage accumulation	Phase I completed in anemic cancer patients; no CRC-specific trials	[89]
	Anti-hemojuvelin antibodies (DISC-0974)	Blocks BMP–SMAD signaling; suppresses hepatic hepcidin production	Rationale: reducing tumor hepcidin could restore antitumor immunity; developed primarily for anemia of chronic disease	Phase I in healthy volunteers; Phase II planned	[150]
**FPN Modulation**					
	miR-194 inhibition	Blocks miR-194-mediated FPN suppression; restores membrane FPN expression and iron efflux	miR-194 drives FPN reduction in advanced CRC; FPN loss correlates with advanced stage and EMT	Early preclinical; conceptual	[151]
	NRF2 modulation	NRF2 transcriptionally activates FPN; modulation influences iron export capacity	NRF2 upregulates FPN alongside SLC7A11 and GPX4; dual role in ferroptosis resistance and iron export	Preclinical	[89]
**Ferroptosis Inducers**					
	Erastin	Inhibits SLC7A11/system Xc^−^; depletes GSH; inactivates GPX4	Synergizes with oxaliplatin in resistant CRC cells in vitro and in vivo; FBXL5 knockdown enhances sensitivity	Preclinical; poor pharmacokinetics limit clinical translation	[143]
	RSL3	Covalently inhibits GPX4; broadly targets the selenoproteome	Reverses oxaliplatin resistance; combined with HIF-1α inhibitor enhances CD8^+^ T cell infiltration in MSS CRC	Preclinical	[152]
	Sorafenib	Inhibits system Xc^−^ and multiple kinases (RAF/VEGFR/PDGFR); induces ferroptosis	First clinically approved drug shown to induce ferroptosis; nanoformulation with quercetin enhances ferroptotic CRC killing	Approved for HCC/RCC; CRC ferroptosis application preclinical	[153]
	Acevaltrate	Dual inhibition of iron chaperones PCBP1/2 and GPX4 degradation	Efficacy surpasses classical ferroptosis inducers and first-line drugs in CRC animal models; active in CRC organoids	Preclinical (2025); first-in-class agent	[154]
**IRP2 Targeting**					
	Trametinib (MEK inhibitor)	Indirectly suppresses IRP2 expression; reduces TfR1 and labile iron pool	Elevated IRP2 in CRC correlates with reduced overall survival; IRP2 suppression delays tumor growth in organoid and xenograft models	FDA-approved for melanoma; preclinical for CRC IRP2 targeting	[79,80]
**Combination Strategies**					
	Erastin + oxaliplatin	Ferroptosis induction overcomes oxaliplatin resistance by bypassing apoptosis-resistance mechanisms	Enhanced cell death in oxaliplatin-resistant CRC cells in vitro and in vivo; reversed by ferroptosis inhibitor ferrostatin-1	Preclinical	[143]
	RSL3 + HIF-1α inhibitor + anti-PD-1	Ferroptosis combined with hypoxia pathway inhibition and immune checkpoint blockade	Markedly enhanced tumor suppression in MSS CRC; increased CD8^+^ T cell infiltration; converts immunologically cold tumors to hot	Preclinical (2026); addresses unmet need in MSS CRC	[155]
	KRAS G12D inhibitor (MRTX-1133) + ferroptosis inducers	KRAS G12D inhibition sensitizes mutant CRC cells to ferroptosis; synergistic lipid peroxidation	Patient-derived organoid validation; relevant to ~40% of CRC harboring KRAS mutations	Preclinical (2025)	[136]

## Data Availability

No new data were created or analyzed in this study. Data sharing is not applicable to this article.

## Abbreviations

The following abbreviations are used in this manuscript:

4-HNE	4-hydroxynonenal
8-OHdG	8-hydroxy-2-deoxyguanosine
ABCB7	ATP-binding cassette subfamily B member 7
ACSL4	Acyl-CoA synthetase long chain family member 4
AHR	Aryl hydrocarbon receptor
APC	Adenomatous polyposis coli
APE1	Apurinic/apyrimidinic endonuclease 1
CIA	Cytosolic iron–sulfur protein assembly
CIAO1	Cytosolic iron–sulfur assembly component 1
CIAO2B	Cytosolic iron–sulfur assembly component 2B
CIN	Chromosomal instability
CRC	Colorectal cancer
DCYTB	Duodenal cytochrome B
DMT1	Divalent metal transporter 1
EGFR	Epidermal growth factor receptor
FAM96B	Family with sequence similarity 96 member B
FECH	Ferrochelatase
Fe–S	Iron–sulfur
FPN	Ferroportin
FSP1	Ferroptosis suppressor protein 1
FTH1	Ferritin heavy chain 1
GLUT1	Glucose transporter 1
GPX4	Glutathione peroxidase 4
HIF-2α	Hypoxia-inducible factor 2 alpha
HIFs	Hypoxia-inducible factors
IDA	Iron deficiency anemia
IGF2BP2	Insulin-like growth factor 2 mRNA-binding protein 2
IL-6	Interleukin 6
IRP2	Iron-regulatory protein 2
ISC	Iron–sulfur cluster assembly
ISCU	Iron–sulfur cluster assembly enzyme
JAK	Janus kinase
LIP	Labile iron pool
LPI	Labile plasma iron
MDA	Malondialdehyde
miR-194	microRNA-194
MMS19	MMS19 homolog, cytosolic iron–sulfur assembly component
MSI	Microsatellite instability
MZF-1	Myeloid zinc finger 1
NCOA4	Nuclear receptor coactivator 4
NFS1	Cysteine desulfurase NFS1
NOX1	NADPH oxidase 1
NQO1	NAD(P)H quinone dehydrogenase 1
NRF2	Nuclear factor erythroid 2-related factor 2
NTBI	Non-transferrin-bound iron
OGG1	8-oxoguanine DNA glycosylase 1
OR	Odds ratio
PCO	Protein carbonyls
PDH	Pyruvate dehydrogenase
PpIX	Protoporphyrin IX
ROS	Reactive oxygen species
SHP	Serum protein thiol
SLC7A11	Solute carrier family 7 member 11
SLPI	Secretory leukocyte protease inhibitor
STAT3	Signal transducer and activator of transcription 3
STEAP4	Six-transmembrane epithelial antigen of the prostate 4
TFRC	Transferrin receptor gene
TfR1	Transferrin receptor 1
TIBC	Total iron-binding capacity
TME	Tumor microenvironment
TSAT	Transferrin saturation
UIBC	Unsaturated iron-binding capacity
Wnt	Wingless/Integrated
XLSA/A	X-linked sideroblastic anemia with ataxia
